# The importance of drying and grinding samples for determining mobile chromium fractions in polluted river sediments

**DOI:** 10.1007/s10661-019-7727-2

**Published:** 2019-08-20

**Authors:** Marzena Trojanowska, Ryszard Świetlik

**Affiliations:** Department of Environmental Protection, Kazimierz Pulaski University of Technology and Humanities in Radom, Chrobrego 27, 26-600 Radom, Poland

**Keywords:** Chromium, Sequential extraction, River sediment, Sample preparation, Leaching kinetics

## Abstract

A possible impact of sample preparation on the chemical fractionation results is generally underestimated in studies of forms of occurrence of heavy metals in river sediments. Our analysis of the recently published results of sequential extraction of chromium has revealed the effect of sample grinding on the result of determination of mobile chromium fractions in river sediments. This observation has been experimentally verified along with the analysation of potential effect of river sediment drying conditions on chromium distribution pattern. The studies were carried out on river sediments polluted with tannery effluents (Cr, 29.2–233 mg/kg). The determined content of chromium bound to carbonates in powdered samples was 2 to 7 times higher than those in raw river sediment samples. It was shown that the main reason was the different kinetic characteristics of chromium leaching in these sediments. Using the shrinking core model, it was found that diffusion through the “ash layer” was the rate-controlling step during the extraction of the carbonate fraction of chromium. It has been additionally confirmed that common air drying of sediment samples does not affect the results of chemical fractionation of chromium.

The results of our studies are also vital for the assessment of environmental risk posed by river sediments polluted with heavy metals. In the case of sediment samples used in this study, powdering changed the risk category (RAC) from *low risk* to *high risk*. Hence, in order to achieve a realistic assessment of chromium mobility and environmental risk, it is advisable to use raw samples, despite their poorer homogeneity, and thus, lower precision of chemical fractionation results.

**Electronic supplementary material:**

The online version of this article (10.1007/s10661-019-7727-2) contains supplementary material, which is available to authorized users.

## Introduction

Globally, chrome tanning generates ca. 11 million m^3^ yr^−1^ of wastes, which contain about 20 Gg of chromium(III) (Morera et al. 2007). Tannery wastewaters are generally directed into sewage treatment plants; however, they are sometimes discharged directly into water reservoirs without any effective treatment (Pawlikowski et al. 2006).

Chromium entering surface waters tends to accumulate in bottom sediments. It binds to various biogeochemical phases generating forms that vary in mobility, bioavailability and toxicity. Chemical fractionation procedures are commonly applied to evaluate the mobility and bioavailability (Hlavay et al. 2004). However, their operational character is the reason why fractionation results depend not only on the kind of the tested sample and its biogeochemical composition but also on the effectiveness and selectivity of the extraction agents used, and extraction conditions (Filgueiras et al. 2002; Gleyzes et al. 2002; Rao et al. 2008). Moreover, the fractionation results can also be affected by the sample preparation (Rapin et al. 1986; Kersten and Förstner 1987; Zhang et al. 2001; Claff et al. 2010a, b).

In the publications where authors carried out fractionation of the chromium present in river sediment, a great importance was attached to the selection of effective fractionation protocol, while the issue of sample preparation seemed to be underestimated (118 publications registered in the SCOPUS database in the years 2000–2018, Supplementary Material: Table 1S). From among a high number of procedures (e.g. Tessier et al. 1979; Shuman 1985; Kersten and Förstner 1986; Grimalt 1989; Ure et al. 1993; Camponella et al. 1995; Hall et al. 1996, and Gómez-Ariza et al. 2000a), the two most commonly used procedures for chromium fractionation in river sediments are those developed by Tessier et al. (1979) (41% of papers) and BCR (Ure et al. 1993) (51% of papers). The original Tessier procedure and the BCR procedure (as well as its later version: Rauret et al. 1999) involve the investigation of bottom sediment samples that were oven dried (105 °C ± 2 °C) to constant mass and then ground. In the reviewed set of publications, the bottom sediment samples were freeze-dried (12%), air dried at RT (43%) and oven dried (33%) (only in eight cases, the drying temperature reached 100–110 °C). In other publications, the samples were either preserved in a freezer until analysis, or the sample preservation conditions were not given. Considering the most commonly declared investigation aims (distribution of Cr and other elements into mineral phases (98%) and environmental risk assessment (32%)), the authors of some of the investigations most likely assumed that the method of preparation of river sediment samples would not affect their results. However, it is possible to find literature reports which do not support such an approach.

**Fig. 1:**
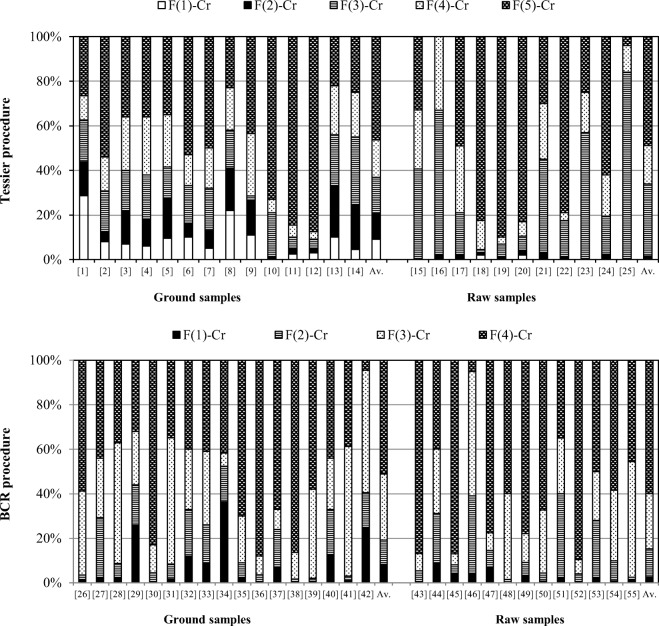
Distribution of chromium fractions in river sediments (published studies: [1] Lin et al. 2018; [2] Lee et al. 2017; [3] Islam et al. 2015; [4 ] Islam et al. 2015a; [5] Islam et al. 2015b; [6] Asa et al. 2013; [7] Dhanakumar et al. 2013; [8] Hejabi and Basavarajappa 2013; [9] Wang et al. 2011; [10] Yu et al. 2010; [11] Giridharan et al. 2010; [12] Rath et al. 2009; [13] Jain et al. 2008; [14] Li et al. 2007; [15] Akele et al. 2016; [16] Chen et al. 2016, [17] Pourabadehei and Mulligan 2015; [18] Fernandes and Nayak 2015; [19] Mayes et al. 2011; [20] Liu et al. 2009, [21] Głosińska et al. 2005; [22] Sáenz et al. 2003; [23] Ho and Egashira 2000; [24] González et al. 2000; [25] Gómez-Ariza et al. 2000b; [26] Xia et al. 2018; [27] Gao et al. 2018; [28] Unda-Calvo et al. 2017; [29] Sayadi et al. 2015; [30] Zhang et al. 2015; [31] Martínez-Santos et al. 2015; [32] Pandey et al. 2015; [33] Pandey et al. 2014; [34] Kumar et al. 2014; [35] Qiao et al. 2013; [36] Yang et al. 2012; [37] Cai et al. 2011; [38] Davutluoglu et al. 2011; [39] Wu et al. 2011; [40] Wang et al. 2010; [41] Kolowski Rodrigues and Formoso 2006; [42] Reis et al. 2005; [43] Liu et al. 2018; [44] Świetlik and Trojanowska 2016; [45] Šestinova et al. 2015; [46] Oyeyiola et al. 2014; [47] Dundar et al. 2013; [48] Roig et al. 2013; [49] Dundar et al. 2012; [50] Nemati et al. 2011; [51] Priadi et al. 2011; [52] Varejão et al. 2011; [53] Yan et al. 2010; [54] Lesven et al. 2009; [55] Arias et al. 2008)

Mobile and bioavailable fractions (water soluble, exchangeable and bound to carbonates) are considered to be the most sensitive to sample preparation conditions (Bordas and Bourg 1998; Filgueiras et al. 2002; Gleyzes et al. 2002). The contact of anaerobic sediments with oxygen may lead to an increase in the fraction of metals associated with oxides and hydroxy-oxides of Fe(III) and Mn(III/IV), as well as a decrease in the share of ion-exchange fraction or carbonate fraction (Rapin et al. 1986; Bordas and Bourg 1998; Rao et al. 2008). Chromium redistribution involving a decrease in the share of mobile fractions was observed by Zhang et al. (2001). Grinding of samples may be another factor that affects the distribution of heavy metals (Claff et al. 2010a). In the reviewed 118 studies, the share of mobile and bioavailable fractions for ground samples is usually several times higher than for raw samples, regardless of the chemical fractionation procedure used (Fig. 1). When using the Tessier procedure, the average share of the ion-exchange and carbonate chromium fractions in ground sediment samples equals 20.6% which is over 12 times higher than in raw sediment samples (1.7%). Smaller differences can be observed in the studies employing the BCR procedure. The ion-exchange and carbonate fractions determined in ground sediment samples bind, on average, 8.2% of chromium, i.e. over 3 times more than in raw sediment samples. The results obtained for the ground samples undoubtedly show true chromium distribution in river sediments and are characterized by better precision, but their basic shortcoming was the fact that they did not reflect the real behaviour of chromium in polluted bed sediments. Overstated results of the determination of mobile fractions may lead to an incorrect evaluation of bioavailability and mobility of chromium present in river sediments resulting in an inadequate prognosis of the environmental risk of chromium pollution, e.g. RAC (*risk assessment code*) (Jain 2004; Hlavay et al. 2004; Claff et al. 2010a, b).

The aim of our research was to experimentally verify these observations from the literature survey and to explain their causes. To achieve this, we carried out an evaluation of the effect of the thermal conditions of drying and grinding river sediment samples on chemical chromium fractionation results. The studies were carried out on river sediment samples polluted with tannery effluents.

## Materials and methods

### Study area

**Fig. 2:**
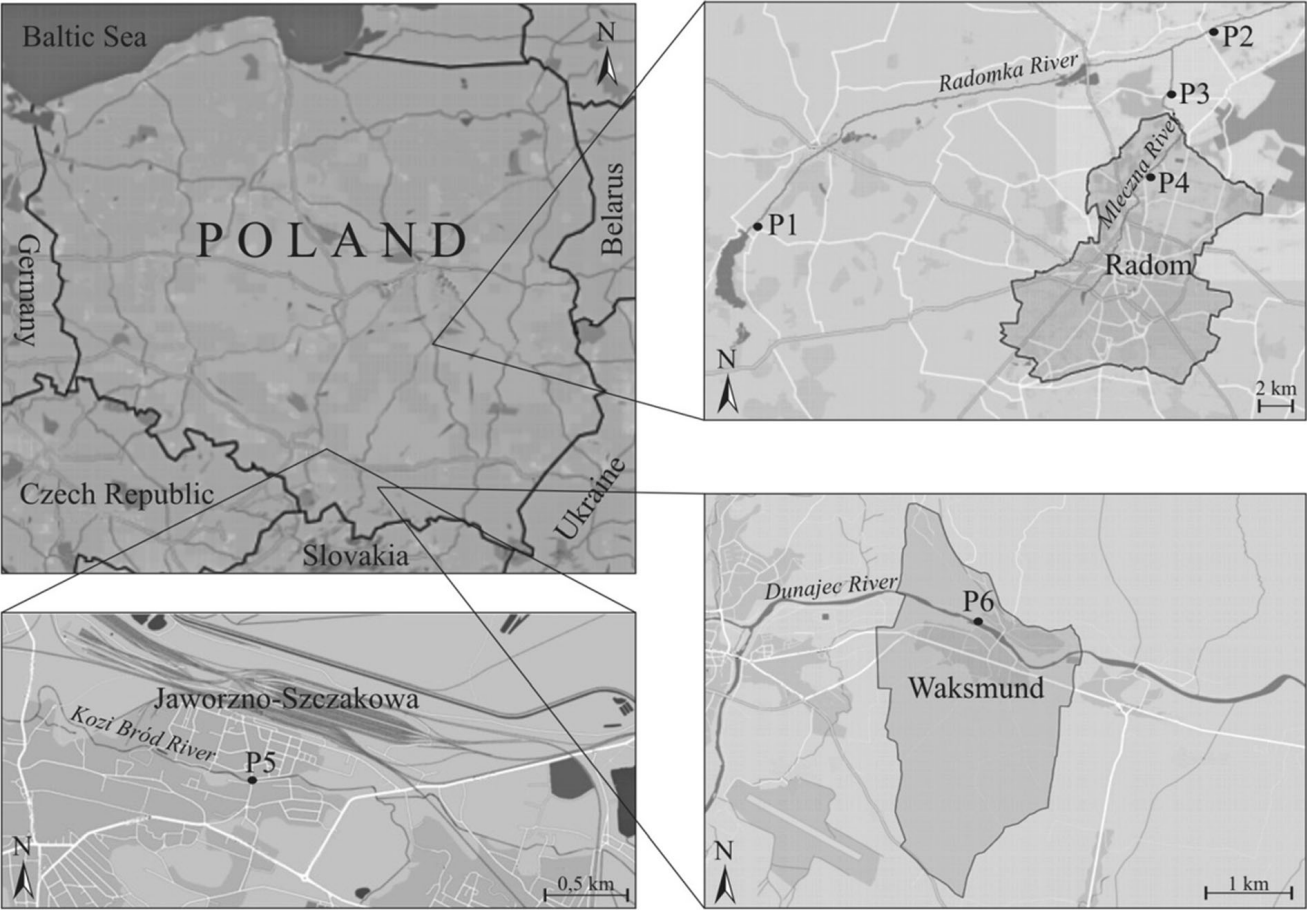
River sediment samples collection sites

The tests were carried out on river sediments from tannery industry regions. The samples were collected in central Poland from the lowland Radomka river (basin area of 2000 km^2^) and its right-bank tributary the Mleczna river (basin area of 350 km^2^) (Fig. 2). Both rivers are quite shallow with sandy beds. The sediment samples from the Radomka river were collected from an unpolluted stretch of the river in Domaniów (P-1) and from site almost 2 km below the tributary of the heavily polluted Mleczna river (Bartodzieje, P-2). The samples of the sediments polluted with chromium from the Mleczna river originated from two sites: Krzewień (P-3) and Firlej (P-4).

In southern Poland, the samples of river sediments polluted with chromium were collected from the Kozi Bród stream (left-bank tributary of the Biała Przemsza river) in Jaworzno-Szczakowa (P-5) and from the Dunajec river (basin area of 6800 km^2^) in Waksmund (P-6).

### Sampling and sample preparation

The sediment samples were collected from the top layer of bottom sediments (up to the depth of 5 cm) in the rivers current area by using a plastic core sediment sampler. After removal of excess water in a vacuum filtration system, the samples were passed through a 2-mm nylon sieve to separate small stones and plant material, and thoroughly homogenized using a plastic spatula. The samples were divided into three parts: the first was frozen to − 20 °C ± 2 °C; the second was dried at room temperature (20 °C ± 2 °C) to constant mass; and the third was dried at 105 °C ± 2 °C to constant mass. Half of each dry sediment sample was ground in a vibratory grinder LMW-S (Testchem) with a corundum grinding vessel until ≤ 120 μm grain fraction was obtained. Then, the prepared samples were stored in tightly closed plastic containers at room temperature in the dark.

The studies were carried out on raw samples stored frozen and air dried at room temperature (RT) (henceforth designated with letters A and B, respectively) and powdered samples which were previously air dried at RT and oven dried at 105 °C (denoted here with letters C and D, respectively). The sediments which were frozen before chemical fractionation were defrosted at room temperature. Wet samples, whose moisture content had previously been determined according to ASTM D2216 (2010), were used in the tests.

### Chemical fractionation—single chemical extraction

The environmentally available metal fraction (pseudo-total metal content, henceforth designated as metal content) in river sediment samples was separated by microwave-assisted digestion of a river sediment sample weighing 0.5 g with 5 cm^3^ of 65% HNO_3_ and 1 cm^3^ of 30% H_2_O_2_ according to the manufacturer’s protocol (Milestone 1992).

### Chemical fractionation—sequential chemical extraction

Metal fractionation was carried out according to the modified Tessier extraction scheme (Table 1). Unlike the original scheme (Tessier et al. 1979), the F(5) fraction was calculated from the difference between the environmentally available metal fraction and the sum of F(1), F(2), F(3) and F(4) fractions. It was called an environmentally persistent fraction to differentiate it from the term residue fraction which is commonly used when sample digestion is carried out in the presence of hydrofluoric acid (Świetlik et al. 2012).

**Table 1:** Operating conditions used in modified Tessier sequential extraction procedure

Stage	Fraction		Reagent	Volume of reagent (mL)	Conditions^a,b^
1	F(1)	Exchangeable	1 M MgCl_2_ (pH 7)	8	Shaking 1 h, RT
2	F(2)	Bound to carbonates	1 M CH_3_COONa, pH 5 (CH_3_COOH )	8	Shaking 5 h, RT
3	F(3)	Bound to Fe/Mn oxides	0.04 M NH_2_OH·HCl/25% CH_3_COOH	20	Shaking 6 h, 96 °C ± 2 °C
4	F(4)	Bound to organic matter and sulphides	30% H_2_O_2_ (pH 2) + 0.02 M HNO_3_ 30% H_2_O_2_ (pH 2) 3.2 M CH_3_COONH_4_/20% HNO_3_	5 + 3 3 5	Shaking 2 h, 85 °C ± 2 °C Shaking 3 h, 85 °C ± 2 °C Shaking 0.5 h, RT
5	F(5)	Environmentally persistent	Calculated as the difference between environmentally available metal content and the sum of the fractions determined	–	–
^a^1.00 g of sample was used ^b^All extracted solutions were separated by centrifugation at 10,000 rpm for 30 min and filtered through a membrane filter Millipore 0.45 μm					

After each stage of extraction, the solid phase was separated from the liquid phase using a centrifuge (MPW 365, Poland) at 10,000 rpm for 30 min. Additionally, the supernatants were filtered through a 0.45-μm syringe filter (Millex-HV).

The quality of the results of chromium fractionation was characterized by the recovery test of the CRM BCR-701 (river sediment). The recoveries were as follows: 96.9% for F(1)-Cr, 95.2% for F(2)-Cr, 104% for F(3)-Cr, 103% for F(4)-Cr and 95.2% for pseudo-total extractable Cr (Supplementary Material: Table 2S).

### Determination of elements

The concentrations of metals in the eluates were determined using an atomic absorption spectrophotometer AAS-3100 Perkin Elmer with flame atomization. Standard solutions that were prepared by appropriate dilution of the stock solution 1000 μg/cm^3^ (BDH) were used to calibrate the device by means of the standard curve method. All measurements were performed in triplicate. The limits of detection were as follows: Cr 0.05 mg/dm^3^, Fe 0.10 mg/dm^3^, Mn 0.06 mg/dm^3^ and Ca 0.09 mg/dm^3^. Lower concentrations of Cr in the eluates were determined by using a Perkin Elmer HGA 600 graphite furnace. The limit of detection was 0.2 μg/dm^3^.

The content of organic carbon in river sediments was determined using the dichromate method (Radojević and Bashkin 1999).

### Studies on the kinetics of chromium release

The leaching processes of F(2)-Cr and F(3)-Cr were performed in the same way as the II and III extraction stages of Tessier scheme (Table 1). In the case of powdered samples, the extractions were stopped after 5, 10, 15, 30, 60, 90, 120, 180, 240, 300 and 360 min, while the raw sediment samples were extracted for 1, 2, 3, 5, 10, 24, 48, 72 and 120 h. The leaching process of each sediment sample was conducted for three subsamples. Chromium was determined as stated in the section “Determination of elements.”

### Chromium pollution assessment of sediment samples

The geoaccumulation index (*I*_geo_) and risk assessment code (RAC) were applied to evaluate the degree of chromium pollution of the sediment samples. The geoaccumulation index was calculated according to the following formula (Müller 1969):

$$ {I}_{\mathrm{geo}}={\log}_2\frac{C}{1.5\cdotp B} $$

where *C* and *B* are content of Cr in a sediment sample and background, respectively. According to *I*_geo_, the samples were classified as follows: *I*_geo_ ≤ 0 pollution free; 0 < *I*_geo_ ≤ 1 slightly polluted; 1 < *I*_geo_ ≤ 2 moderately polluted; 2 < *I*_geo_ ≤ 3 moderate to strongly polluted; 3 < *I*_geo_ ≤ 4 strongly polluted; 4 < *I*_geo_ ≤ 5 strongly to extremely polluted; and *I*_geo_ > 5 extremely polluted (Müller 1969).

The risk assessment code (RAC) is a common ecological risk evaluation index which is calculated from the formula (Perin et al. 1985):

$$ \mathrm{RAC}=\frac{\mathrm{F}(1)-\mathrm{Cr}+\mathrm{F}(2)-\mathrm{Cr}}{\mathrm{C}}\cdotp 100\% $$

where F(1)-Cr and F(2)-Cr are the content of exchangeable and carbonate-bound chromium fractions, respectively, and C is the chromium content in sediment samples. According to RAC index, the samples were classified as follows: ≤ 1% no risk; 1% < RAC ≤ 10% low risk; 10% < RAC ≤ 30% moderate risk; 30% < RAC ≤ 50% high risk; and 50% < RAC very high risk.

## Results and discussion

### Sample characteristics

The river sediments selected for this investigation were, to a varying degree, contaminated with chromium (Table 2). Relatively, high Cr contents were found in sediment samples from the Dunajec river (P-6A, 233 ± 15 mg/kg) and from the Mleczna river (P-4A, 204 ± 23 mg/kg). Apart from the unpolluted sediment sample P-1A (2.48 ± 0.40 mg/kg), Cr content in the other sediment samples exceeded considerably the average chromium content in river sediments in Poland (5 mg/kg) (Bojakowska and Sokołowska 1998). Using the geoaccumulation index to assess the pollution level of chromium, we were able to classify bed sediments of sample P-1A as unpolluted, sample P-5A as strongly polluted, and P-3A, P-4A and P-6A samples as strongly to extremely polluted (Müller 1969; Dundar et al. 2013).

**Table 2:** Content of chromium and matrix elements of geochemical phases in river sediment samples (mean value (*n* = 3) ± standard deviation, *P* = 95%)

Sample	Cr (mg/kg)	*I*_geo_ class	Ca (mg/kg)	Fe (mg/kg)	Mn (mg/kg)	C-org. (mg/kg)
P-1A	2.48 ± 0.40	0	380 ± 53	2384 ± 130	54.8 ± 4.1	0.42 ± 0.03
P-2A	29.2 ± 3.6	2	912 ± 75	2865 ± 140	134 ± 14	1.2 ± 0.01
P-3A	121 ± 9	5	1890 ± 90	2288 ± 152	31.9 ± 6.3	3.0 ± 0.02
P-4A	204 ± 23	5	3090 ± 290	1320 ± 140	26.7 ± 3.2	1.4 ± 0.02
P-5A	83.8 ± 8.2	4	1150 ± 40	2880 ± 140	34.7 ± 1.1	3.3 ± 0.04
P-6A	233 ± 15	5	10,450 ± 190	11,960 ± 460	293 ± 26	7.8 ± 0.06

The samples of river sediments were also characterized by a varying content of elements considered the main components of geochemical phases binding chromium (matrix elements). The samples that were highly polluted with chromium were also enriched with Ca (carbonate fraction), max. 10450 ± 190 mg/kg; Fe and Mn (oxide fraction), max. 11960 ± 460 mg/kg and 293 ± 26 mg/kg, respectively; and C-org., max. 7.8 mg/kg (Table 2).

### Evaluation of the effect of sample preparation conditions

The studies of the effects of preparation of river sediment samples on the results of fractionation of anthropogenic chromium, particularly the determination of its most mobile and bioavailable fractions, were focused on the role of two factors: the thermal conditions of the sample drying and the grinding of the samples. Their selection was a result of a detailed analysis of articles published within the last two decades (Supplementary Material: Table 1S).

The effects of air drying and oven drying on chromium fractionation results were evaluated in relation to deeply frozen raw sediment samples, whereas the effect of sediment grinding on these results was evaluated by examining powdered samples ≤ 120 μm and raw sediment samples.

The assessment of the statistical significance between the determined contents of chemical fractions of chromium in differently prepared samples was verified by means of the Student’s *t* test for the significance level of *α* = 0.05 and the number of degrees of freedom *f* = 4 in a two-tailed test.

#### The effect of air drying

**Fig. 3:**
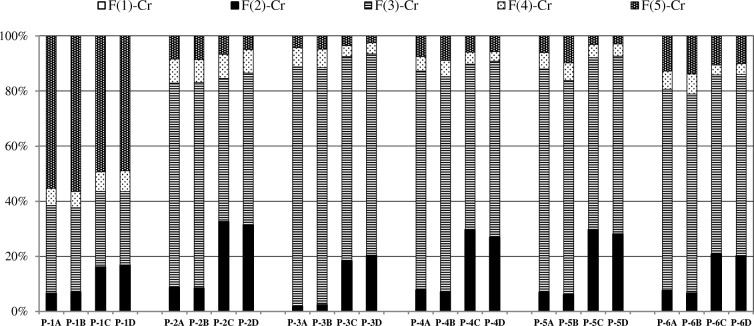
Effect of sediment samples preparation on the distribution pattern of chromium: (**A**) frozen raw sample; (**B**) air-dried raw sample; (**C**) air-dried ground sample; (**D**) oven-dried ground sample

The exposure of sediment samples to atmospheric oxygen during the drying process at 20 °C and 105 °C basically did not result in a change in chromium distribution (samples A vs B and C vs D in Fig. 3). The determined content of the environmentally available chromium and the contents of other chemical fractions of chromium in frozen samples and air dried samples did not show statistically significant differences (Supplementary Material: Table 3S); the calculated values of *t*_*c*_ were generally considerably lower than *t*_0.05_ = 2.776. Air drying did not affect the content of Ca associated with the carbonate fraction either (Supplementary Material: Table 4S). The behaviour of Fe and Mn was slightly different but remained in accordance with earlier reported studies (Rapin et al. 1986; Bordas and Bourg 1998; Rao et al. 2008). The contents of F(2)-Fe in the air dried raw samples were lower than in the frozen samples by 15–25%. An exception was the P-6A sample (the Dunajec river) in which the content of F(2)-Fe decreased considerably after air drying—from 439 to 207 mg/kg. A decrease in the content of F(2)-Mn was on average more balanced, 20% in all air dried raw samples. Simultaneously, an increase in the contents of the F(3)-Fe and F(3)-Mn fractions was observed. The contents of F(3)-Mn determined in the air-dried sediment samples differed significantly from the values determined for reference samples (P-1/6A samples), while change in F(3)-Fe contents was too small to be classified as statistically significant (Supplementary Table 4S). Thus, the results showed that a partial transformation of F(2)-Mn to F(3)-Mn and F(2)-Fe to F(3)-Fe during the drying of sediment samples may not have an effect on chromium distribution between carbonate and Fe/Mn-oxide fractions (Supplementary Material: Table 3S).

#### The effect of grinding

Grinding of sediment samples resulted in a marked change in chromium fractionation results (samples B vs C, Fig. 3). The content of F(2)-Cr determined in powdered samples was several times higher than in the raw samples—from twofold in a sample unpolluted with chromium (P-1A) to sevenfold in chromium polluted samples. The contents of F(3)-Cr and F(4)-Cr, determined in subsequent stages of sequential extraction, were appropriately lower compared with raw samples (Supplementary Material: Table 3S). Analogous tendencies can be observed in the case of the determined contents of F(2)-Fe and F(3)-Fe, as well as F(2)-Mn and F(3)-Mn (Supplementary Table 4S). The determination of lower carbonate fraction contents in raw samples may be explained by the presence of barriers in the structure of raw sediment particles preventing penetration by an extracting agent of the sites where chromium was bound to carbonates. Such a possibility was mentioned in earlier publications, where mineral particles are covered completely or partially by the coatings or films of Fe/Mn oxyhydroxides and organic matter (e.g. Rendell et al. 1980; Förstner 1984), but the analytical consequences of this phenomenon have not yet been investigated.

By adapting the classification of mineral grains according to their accessibility to leach solutions (Ghorbani et al. 2011), it can be assumed that chromium associated with a given geochemical phase may be variously distributed in river sediment particles:

- On the surface, directly exposed to a leaching reactant,
- Inside the particle structure, available for the leaching reactant through the pores and cracks,
- Inside the particle structure which becomes exposed to the leaching reactant after dissolution of a more resistant geochemical phase,
- Inside the primary particle structure, not connected to pores or fissures, or when the spreading of either pores or fissures does not extend to the particle surface.

In each case, river sediment grinding should lead to higher direct availability of a leaching reactant and thus increase the extraction efficiency. Only chromium of the (a) and (b) types can be released at the early stages of sequential extraction of raw sediment samples. Chromium of the (c) type can be released at a later stage ensuring dissolution of a more resistant geochemical phase than the one it is bound to. The release of chromium of the (d) type can take place at the stage of complete dissolution of the matrix–determination of residue fraction. The proposed approach is well illustrated by the results of the kinetic studies on the release of the F(2)-Cr and F(3)-Cr fractions.

### Kinetics analysis

**Fig. 4:**
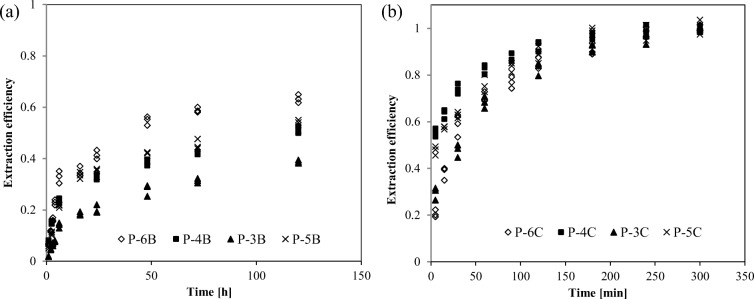
The extraction course of F(2)-Cr from raw samples (**a**) and from ground samples (**b**) of river sediments

The leaching curves of carbonate fraction of chromium for raw and powdered samples differ considerably (Fig. 4). The process of chromium extraction from raw samples was long duration hour scale vs minute scale for powdered samples. In both cases, the leaching process can be divided into two phases: *instant release* referring to chromium directly exposed to an extractant (chromium type a) and *slower release*—probably as a result of resistance associated with diffusion through the pores and cracks (chromium type b). In raw samples, not more than 5% of total F(2)-Cr was rapidly dissoluted, whereas in the case of powdered samples, 20% to nearly 55% of total F(2)-Cr was dissoluted in the instant release phase. Grinding of sediment samples also had a “levelling effect” on the course of chromium leaching. The structure of particles in raw sediment samples permanently determined the course of the leaching curves, whereas the initial differences in chromium leaching efficiency for powdered samples were disappearing with extraction time (Fig. 4).

**Fig. 5:**
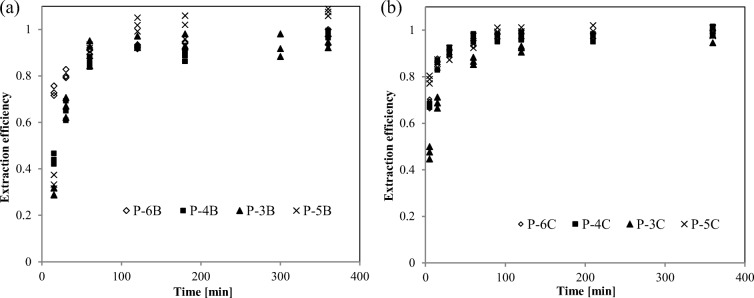
The extraction course of F(3)-Cr from raw samples (**a**) and from ground samples (**b**) of river sediments

The curves of chromium leaching with a more reactive reactant (stage III of Tessier scheme) have a definitely flatter profile—in raw samples from 30 to 75% of total F(3)-Cr and in powdered samples from 40 to 80% of total F(3)-Cr were rapidly dissoluted (Fig. 5). Compared with stage II of chromium extraction, the resistance diffusion is of considerably less importance for the course of chromium extraction. It was shown that the complete release of chromium bound to Fe/Mn oxides was achieved after about 100 min in raw and powdered samples. The small differences in the leaching curves for the F(3)-Cr fraction confirmed our hypothesis that a film of Fe/Mn oxides partly, but effectively, encapsulated chromium species bound to carbonates during stage II chemical fractionation.

As the second stage of the Tessier procedure turned out to be critical for the fractionation pattern of chromium, the experimental data were also analyzed kinetically by using the shrinking core model (SCM), which is widely used to model fluid-solid reactions such as the leaching of metals from minerals (Georgiou and Papangelakis 1998; Abdel-Aal 2000; Seyed Ghasemi and Azizi 2017). In this work, the equations of SCM express the relationship between the fraction of chromium release (*x*) and time (*t*) (min), *k* being the apparent rate constant (h^−1^) (Table 3). All the model equations displayed a reasonable correlation with the experimental data. However, the best fit was obtained for the ash layer diffusion model: *R*^2^ = 0.9102—0.9796 (raw samples) and *R*^2^ = 0.9477—0.9808 (powdered samples). Hence, the diffusion of the extractant or chromium species through the “ash layer” was most likely the rate-controlling step during the extraction of the carbonate fraction of chromium. It should be emphasized that this model equation is very similar to the expression used to describe the leaching kinetics of porous solids according to the grain model (Georgiou and Papangelakis 1998). Without deciding which model represents better the leaching kinetics physically, it is quite likely that the diffusion resistance attributed by us to the Fe/Mn-oxide coatings insulating F(2)-Cr grains in sediment particles, is the reason why the mean rate constant of chromium liberation from raw samples was by two orders of magnitude lower than the one for powdered samples (0.00082 ± 0.00068 h^−1^ and 0.21 ± 0.02 h^−1^, respectively). The coefficients of variation (CV) of these rate constants (82.5% and 9.5%) illustrate very well the “levelling effect” of a leaching course caused by grinding of various river sediment samples.

**Table 3:** Values of regression coefficients (*R*^2^) and *k* values of reaction models for leaching carbonate fraction of chromium from raw (B) and ground (C) river sediment samples

Sample	Model								
	Film diffusion control (small particles)			Chemical reaction control (sphere particles)			Ash layer diffusion control (sphere particles)		
	Equation	*k* (h^−1^)	*R*^2^	Equation	*k* (h^−1^)	*R*^2^	Equation	*k* (h^−1^)	*R*^2^
P-3B	K·t = 1 − (1 − *x*)^2/3^	0.0021	0.8675	K·t = 1 − (1 − *x*)^1/3^	0.0014	0.8758	K·t = 1 − 3(1 − x)^2/3^ + 2(1 − x)	0.00050	0.9796
P-4B		0.0024	0.8553		0.0012	0.8813		0.00090	0.9676
P-5B		0.0027	0.7778		0.0015	0.8041		0.00010	0.9297
P-6B		0.0035	0.7815		0.0021	0.8108		0.0017	0.9102
		–			-			*k*_._ = 0.00082 ± 0.00068 CV = 82.5%	
P-3C		0.20	0.9350		0.16	0.9673		0.20	0.9808
P-4C		0.16	0.8956		0.14	0.9403		0.21	0.9477
P-5C		0.19	0.9470		0.17	0.9539		0.23	0.9581
P-6C		0.21	0.8559		0.16	0.9115		0.19	0.9548
		–			-			*k* = 0.21 ± 0.02 CV = 9.5%	

## Conclusions

The grinding of the samples intended for chemical fractionation can result in the overestimation of the contents in the most mobile chromium fractions of river bed sediments polluted by tannery effluents. The determined content of chromium bound to carbonates in powdered samples was 2 to 7 times higher than those in raw river sediment samples. The determined content of the oxide and organic fractions of chromium was appropriately lower. It has been shown that different kinetic characteristics of the leaching of this chromium fraction were the reason for different results of determination of chromium bound to carbonate in raw samples and in ground samples. By applying the shrinking core model, it was found that the diffusion through the “ash layer” was the rate controlling step during the extraction of the carbonate fraction of chromium from both raw and powdered samples. As there were no similar differences in leaching courses at later stages of sequential extraction, it was concluded that Fe/Mn-oxide coatings hinder or prevent penetration of the sites containing chromium bound to carbonates by an acetate extractant.

It has also been confirmed that the commonly used method of air drying sediment samples does not affect the results of determination of mobile fractions of chromium, although if the object of the study were anoxic sediments, verification of the statement might be recommended.

**Fig. 6:**
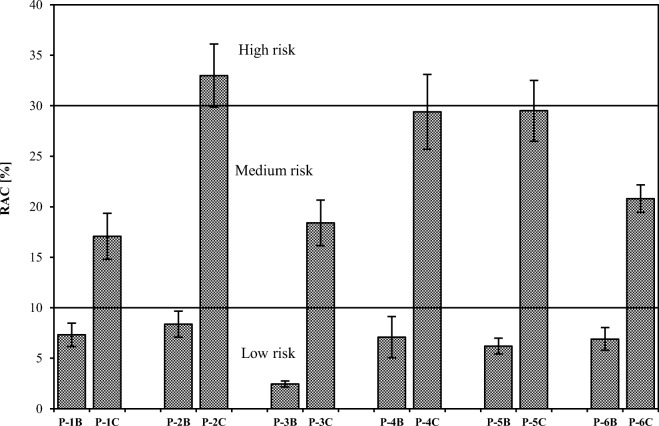
Comparison of risk assessment code (RAC) values for raw (B) and ground (C) sediment samples

The results of our studies are also of great importance for the assessment of the environmental risk posed by the river sediments polluted with heavy metals. In the case of river sediment samples used in this study, powdering changed the risk category from *low risk* to even *high risk* (Fig. 6). Hence, in order to achieve real assessment of chromium mobility and environmental risk, it is necessary to use raw samples, despite their poorer homogeneity and therefore lower precision of chemical fractionation results.

## Electronic supplementary material

ESM 1 (DOCX 80 kb)
