# Supplementary material for: The importance of drying and grinding samples for determining mobile chromium fractions in polluted river sediments
Source: Environ Monit Assess. 2019 Aug 20;191(9):578. doi: 10.1007/s10661-019-7727-2 (PMC6702188; doi:10.1007/s10661-019-7727-2)
Supplement: Supplementary file 1 — (DOCX 80 kb) [file 10661_2019_7727_MOESM1_ESM.docx]

SUPPLEMENTARY MATERIAL TO:

**The importance of drying and grinding of samples for determining mobile chromium fractions in polluted river sediments**

Marzena Trojanowska and Ryszard Świetlik^[[1]](#footnote-1)^

Kazimierz Pulaski University of Technology and Humanities in Radom, Department of Environmental Protection, Chrobrego 27, 26-600 Radom, Poland

**Page 15 Supporting Material**

**Table 1S** Chemical fractionation of chromium and other heavy metals in studies on polluted river sediments. Review of literature 2000-2018

**Table 2S** The results of determined and certified values for certified reference material (BCR 701) mean (mg/kg dry weight ± SD), n = 6

**Table 3S** Effect of sediment sample preparation on the determination results of chemical fractions of chromium (A – frozen raw sample; B - air dried raw sample; C – air dried ground sample; D - oven dried ground sample). Critical value of Student's *t* distribution with *4* degrees of freedom *t* = 2.776, (two-tailed test, P = 95*%*)

**Table 4S** Effect of sediment sample preparation on the determination results of carbonate and oxide fractions of Ca, Fe and Mn (A – frozen raw sample; B - air dried raw sample; C – air dried ground sample; D - oven dried ground sample). Critical value of Student's *t* distribution with *4* degrees of freedom *t* = 2.776 (two-tailed test, P = 95%)

**Table 1S** Chemical fractionation of chromium and other heavy metals in studies on polluted river sediments. Review of literature 2000-2018

| **No** | **Reference** | **Elements studied** |
| --- | --- | --- |
| 1 | Liu, X., Jiang, J., Yan, Y., Dai, Y.Y., Deng, B., Ding, S., Su, S., Sun, W., Li, Z., & Gan, Z. (2018). Distribution and risk assessment of metals in water, sediments, and wild fish from Jinjiang River in Chengdu, China. *Chemosphere*, 196, 45-52. DOI 10.1016/j.chemosphere.2017.12.135 | As, Cd, Co, **Cr**, Cu, Mn, Mo, Ni, Pb, Sb, Se, Sn, Tl, Zn, and V |
| 2 | Minu, A., Routh, J., Dario, M., Bilosnic, M., Kalén, R., Klump, J. V., & Machiva, J. F. (2018). Temporal and spatial distribution of trace metals in the Rufiji delta mangrove, Tanzania. *Environmental Monitoring and Assessment*, 190, 336. DOI 10.1007/s10661-018-6707-2 | As, Cd, **Cr**, Cu, Ni, Pb, and Zn |
| 3 | Xia, F., Qu, L., Wang, T., Luo, L., Chen, H., Dahlgren R. A., Zhang, M., Mei, K., & Huang, H. (2018). Distribution and source analysis of heavy metals pollutants in sediments of a rapid developing urban river system. *Chemosphere,* 207, 218-223. DOI 10.1016/j.chemosphere.2018.05.090 | Cd, **Cr**, Cu, Pb, and Zn |
| 4 | Gurung**,** B., Race, M., Fabbricino, M., Kominkova, D., Libralato, G., Siciliano A., & Guida M. (2018). Assessment of metal pollution in the Lambro Creek (Italy). *Ecotoxicology and Environmental Safety*, 148, 754-762. | **Cr**, Cu, Ni, Pb, and Zn |
| 5 | Gao, L., Wang, Z., Li, S., & Chen, J. (2018). Bioavailability and toxicity of trace metals (Cd, Cr, Cu, Ni, and Zn) in sediment cores from the Shima River, South China. *Chemosphere,* 192, 31-42. DOI 10.1016/j.chemosphere.2017.10.110 | Cd, **Cr**, Cu, Ni, and Zn |
| 6 | Xie, Y., Lu, G., Yang, C., Lu, Q., Chen, M., Guo, C., Dang, Z. (2018). Mineralogical characteristics of sediments and heavy metals mobilization along a river watershed affected by acid mine drainage. PLOS One, 13, 1:e0190010. DOI 10.1371/journal.pone.0190010 | As, Cd, **Cr**, Cu, Mn, Ni, Pb, and Zn, |
| 7 | Lin, J., Zhang, S., Liu, D., Yu, Z., Zhang, L., Cui, J., Xie, K., Li, T., & Fu, C. (2018). Mobility and potential risk of sediment-associated heavy metal fractions under continuous drought–rewetting cycles. *Science of the Total Environment*, 625, 79-86. DOI 10.1016/j.scitotenv.2017.12.167 | Cd, **Cr,** Cu, Mn, and Pb |
| 8 | Unda-Calvo, J., Martinez-Santos, M., & Ruiz-Romera, E. (2017). Chemical and physiological metal bioaccessibility assessment in surface bottom sediments from the Deba River urban catchment: Harmonization of PBET, TCLP and BCR sequential extraction methods. *Ecotoxicology and Environmental Safety*, 138, 260-270. DOI 10.1016/j.ecoenv.2016.12.029 | **Cr,** Cu, Fe, Mn, Ni, Pb, and Zn |
| 9 | Jun, R., Zhen, S., Ling, T., & Jianxiu H. (2017). Speciation and contamination assessment of metals in the sediments from the Lanzhou Section of the Yellow River, China. *Environment Protection Engineering,* 43, 3, 113-124. | Cd, Cr, Cu, Fe, Mn, Ni, Pb, and Zn |
| 10 | Lee, P. K., Kang M. J., Yu, S., Ko, K. S., Ha, K., Shin, S. C., & Park J. H. (2017). Enrichment and geochemical mobility of heavy metals in bottom sediment of the Hoedong reservoir, Korea and their source apportionment. *Chemosphere,* 184, 74-85. DOI 10.1016/j.chemosphere.2017.05.124 | Cd, **Cr**, Cu, Ni, Pb, and Zn |
| 11 | Zhang, C., Shan, B., Tang, W., Dong, L., Zhang, W., & Pei, Y. (2017). Heavy metal concentrations and speciation in riverine sediments and the risks posed in three urban belts in the Haihe Basin. *Ecotoxicology and Environmental Safety*, 139, 263-271. DOI 10.16/j.ecoenv.2017.01.047 | **Cr**, Cu, Ni, Pb, and Zn |
| 12 | Zhao, C. P., Zhou, X. J., Dong, K., Fu, J., Zhu, D. D., An, S. Q., & Zhu, H. L. (2017). Potential ecological risk and speciation analysis of heavy metals in sediments from the Jialu River, China. *International Journal of Environment and Pollution,* 61, 1, 72-88. | Cd, Co, **Cr**, Cu, Mn, Ni and Zn |
| 13 | Chen, Y., Dong, B., & Xin, J. (2017). Occurrence and fractionation of Cr along the Loushan River affected by a chromium slag heap in East China. *Environmental Science and Pollution Research*, 24, 15655-15666. DOI 10.1007/s11356-017-9200-5 | **Cr** |
| 14 | Vosoogh, A., Saeedi, M., & Lak, R. (2017). Metal fractionation and pollution risk assessment of different sediment sizes in three major southwestern rivers of Caspian Sea. Environmental Earth Sciences, 76, 292. DOI 10.1007/s12665-017-6603-z | Cd, Co, **Cr**, Cu, Ni and V |
| 15 | Akele, M. L., Kelderman, P., Koning, C. W., & Irvine, K. (2016). Trace metal distributions in the sediments of the Little Akaki River, Addis Ababa, Ethiopia. Environmental Monitoring *and* Assessment, 188, 389. DOI 10.1007/s10661-016-5387-z. | Cd, Co, **Cr,** Cu, Mn, Ni, and Zn |
| 16 | Liu, H., Liu, G., Wang, J., Yuan, Z., & Da, Ch. (2016). Fractional distribution and risk assessment of heavy metals in sediments collected from the Yellow River, China. *Environmental Science and Pollution Research*, 23, 11076-11084. DOI 10.1007/s11356-016-6291-3 | **Cr**, Mn, Ni, Pb and Zn |
| 17 | Sakan, S., Popović, A., Anđelković, I., & Ðorđević D. (2016). Aquatic sediments pollution estimate using the metal fractionation, secondary phase enrichment factor calculation, and used statistical methods. *Environmental Geochemistry and Health*, 38, 855–867. DOI 10.1007/s10653-015-9766-0 | Cd, **Cr**, Cu, Fe, Mn, Ni, Pb, Zn and V |
| 18 | Świetlik, R. & Trojanowska, M. (2016). Mobility of chromium and its chemical fractions in river sediment polluted by tannery effluents (Poland). *Soil and Sediment Contamination: An International Journal*, 25, 3, 266-278. DOI 10.1080/15320383.2016.1130686 | **Cr** |
| 19 | Tatone, L. M., Bilos, C., Skorupka, C. N., & Colombo, J. C. (2016). Comparative approach for trace metal risk evaluation in settling particles from the Uruguay River, Argentina: enrichment factors, sediment quality guidelines and metal speciation. Environmental Earth *Sciences*, 75, 575. DOI 10.1007/s12665-016-5265-6 | Cu, **Cr**, Fe, Mn, Ni, Pb and Zn |
| 20 | Chen, W. F., Zhang, J., Zhang, X., Wang, W., & Li, Y. (2016). Investigation of heavy metal (Cu, Pb, Cd, and Cr) stabilization in river sediment by nano-zero-valent iron/activated carbon composite. Environmental Science *and* Pollution *Research,* 23, 1460-1470. DOI 10.1007/s11356-015-5387-5 | Cd, **Cr**, Cu and Pb |
| 21 | Sayadi, M. H., Rezaei, M. R., & Rezaei, A. (2015). Fraction distribution and bioavailability of sediment heavy metals in the environment surrounding MSW landfill: a case study. Environmental Monitoring *and* Assessment, 187, 4110. DOI 10.1007/s10661-014-4110-1 | Cd, **Cr**, Cu, Ni, Pb and Zn |
| 22 | Zhang, L., Liao, Q., Shao, S., Zhang, N., Shen, Q., & Liu, C. (2015). Heavy Metal Pollution, Fractionation, and Potential Ecological Risks in Sediments from Lake Chaohu (Eastern China) and the Surrounding Rivers. The International Journal *of* Environmental Research *and* Public Health*,* 12, 14115-14131. DOI 10.3390/ijerph121114115 | Cd, **Cr**, Cu, Ni, Pb and Zn |
| 23 | Šestinova, O., Findoráková, L., Hančul’ák, J., Šestinova, L. (2015). Study of metal mobility and phytotoxicity in bottom sediments that have been influenced by former mining activities in Eastern Slovakia. *Environmental Earth Sciences*, 74, 6017-6025. DOI 10.1007/s12665-015-4625-y | As, Cd, **Cr**, Cu, Hg, Ni, Pb, Sb, and Zn |
| 24 | Islam, M. S., Ahmed, M. K., Habibullah-Al-Mamun, M., & Islam, M. K. (2015). Geochemical Speciation and Risk Assessment of Heavy Metals in Sediments of a River in Bangladesh. *Soil and Sediment Contamination*, 24(6), 639-655. DOI: 10.1080/15320383.2015.997869 | As, Cd, **Cr**, Cu, Ni, and Pb |
| 25 | Venkatramanan, S., Chung, S. Y., Ramkumar, T., & Selvam, S. (2015). Environmental monitoring and assessment of heavy metals in surface sediments at Coleroon River Estuary inTamil Nadu, India. Environmental Monitoring *and* Assessment*,* 187, 505. DOI 10.1007/s10661-015-4709-x | Co, **Cr**, Cu, Fe, Mn, Ni, Pb and Zn |
| 26 | Martínez-Santos, M., Probst, A., García-García, J., & Riuz-Romera, E. (2015). Influence of anthropogenic inputs and a high-magnitude flood event on metal contamination pattern in surface bottom sediments from the Deba River urban catchment. *Science of the Total Environment*, 514, 10-25. DOI 10.1016/j.scitotenv.2015.01.078 | **Cr**, Cu, Fe, Mn, Ni, Pb and Zn |
| 27 | Jabłońska-Czapla, M. (2015). Antimony, Arsenic and Chromium Speciation Studies in Biała Przemsza River (Upper Silesia, Poland) Water by HPLC-ICP-MS. *The* International Journal *of* Environmental Research *and* Public Health, 12, 4739-4757. DOI 10.3390/ijerph120504739 | As, **Cr**, and Sb |
| 28 | Islam, M. S., Ahmed, M. K., Raknuzzaman, M., Habibullah-Al-Mamun, M., & Masunaga S. (2015b). Metal speciation in sediment and their bioaccumulation in fish species of three urban rivers in Bangladesh. Archives of Environmental Contamination *and* Toxicology, 68, 92-106. DOI 10.1007/s00244-014-0079-6 | As, Cd, **Cr**, Cu, Ni, and Pb |
| 29 | Pandey, M., Pandey, A. K., Mishra, A., & Tripathi, B. D. (2015). Assessment of metal species in river Ganga sediment at Varanasi, India using sequential extraction procedure and SEM–EDS. *Chemosphere*, 134, 466-474. DOI 10.1016/j.chemosphere.2015.04.047 | Cd, **Cr**, Cu, Fe, Mn, Ni, Pb and Zn |
| 30 | Islam, M. S., Ahmed, M. K., Raknuzzaman, M., Habibullah-Al-Mamun, M., & Islam, M.K. (2015c). Heavy metal pollution in surface water and sediment: A preliminary assessment of an urban river in a developing country. *Ecological Indicators*, 48, 282-291. DOI 10.1016/j.ecolind.2014.08.016 | As, Cd, **Cr**, Cu, Ni and Pb |
| 31 | Pourabadehei, M., & Mulligan, C. N. (2015). Geochemical and physical characteristics of contaminated sediment in a harbour area. Japanese Geotechnical Society Special Publication, The 15^th^ Asian regional Conference on Soil Mechanics and Geotechnical Engineering. DOI 10.3208/jgssp.OTH-19 | As, Cd, **Cr**, Cu, Ni, Pb and Zn |
| 32 | Fernandes, M. C., & Nayak, G. N. (2015). Speciation of metals and their distribution in tropical estuarine mudflat sediments, southwest coast of India. *Ecotoxicology and Environmental Safety,* 122, 68-75. DOI 10.1016/j.ecoenv.2015.07.0216 | Co, **Cr**, Cu, Fe, Mn, Ni and Zn |
| 33 | Oyeyiola, A. O., Davidson, C. M., Olayinka, K. O., & Alo, B. I. (2014). Fractionation and ecotoxicological implication of potentially toxic metals in sediments of three urban rivers and the Lagos Lagoon, Nigeria, West Africa. Environmental Monitoring *and* Assessment, 186, 7321–7333. DOI 10.1007/s10661-014-3929-9 | Cd**, Cr**, Cu, Pb |
| 34 | Pandey, M., Tripathi, S., Pandey, A. K., & Tripathi, B. D. (2014). Risk assessment of metal species in sediments of the river Ganga. *Catena*, 122, 140-149. DOI 10.1016/j.catena.2014.06.012 | Cd, Co, **Cr**, Cu, Fe, Mn, Ni, Pb and Zn |
| 35 | Sungur, A., Soylak, M., Yilmaz, S., & Özcan, H. (2014). Determination of heavy metals in sediments of the Ergene River by BCR sequential extraction method. *Environmental Earth Sciences,* 72, 3293-3305. DOI 10.1007/s12665-014-3233-6 | Cd, Co, **Cr**, Cu, Mn, Ni, Pb and Zn |
| 36 | Dundar, M. S., Altundag, H., Eyupoglu, V., Keskin, C. S., & Tutunoglu, Ç. (2013). Sequential extraction speciation of heavy metals in sediments based on grain size. *Fresenius Environmental Bulletin,* 22 (11), 3184-3191 | Cd, Co, **Cr**, Cu, Fe, Mn, Ni, Pb and Zn |
| 37 | Kumar, R., Rani, M., Gupta, H., & Gupta, B. (2014). Trace metal fractionation in water and sediments of an urban river stretch. *Chemical Speciation & Bioavailability*, 26,4, 200-209. DOI 10.3184/095422914X14142369069568 | **Cr**, Cu, Ni and Pb |
| 38 | Copaja, S. V., Molina, X., & Tessada, R. (2014). Determination of heavy metals in Choapa River sediments using BCR sequential extraction procedure. *Journal of the Chilean Chemical Society*, 59, 1, 2353-2358. | Al, **Cr**, Cu, Fe, Mn, Ni, Pb and Zn |
| 39 | Roig, N., Sierra, J., Ortiz, J. D., Merseburger, G., Schuhmacher, M., Domingo, J. L., & Nadal, M. (2013). Integrated study of metal behavior in Mediterranean stream ecosystems: A case-study. *Journal of Hazardous Materials*, 263P, 122-130. DOI 10.1016/j.jhazmat.2013.07.051 | As, Cd, Co, **Cr**, Cu, Hg, Ni, Pb and Zn |
| 40 | Canuto, F. A. B., Garcia, C. A. B., Alves, J. P. H., & Passos, A. E. (2013). Mobility and ecological risk assessment of trace metals in polluted estuarine sediments using a sequential extraction scheme. Environmental Monitoring *and* Assessment, 185, 6173-6185. DOI 10.1007/s10661-012-3015-0 | Cd, **Cr**, Cu, Ni, Pb and Zn |
| 41 | Asa, S. Ch., Rath, P., Panda, U. C., Parhi, P. K., & Bramha, S. (2013). Application of sequential leaching, risk indices and multivariate statistics to evaluate heavy metal contamination of estuarine sediments: Dhamara Estuary, East Coast of India. Environmental Monitoring *and* Assessment*,* 185, 6719-6737. DOI 10.1007/s10661-013-3060-3 | Cd, Co, **Cr**, Cu, Fe, Mn, Ni, Pb and Zn |
| 42 | Dhanakumar, S., Murthy, K. R., Solaraj, G., & Mohanraj, R. (2013). Heavy-metal fractionation in surface sediments of the Cauvery River Estuarine Region, Southeastern Coast of India. Archives of Environmental Contamination *and* Toxicology, 65, 14-23. DOI 10.1007/s00244-013-9886-4 | **Cr**, Cu, Fe, Mn, Ni and Pb |
| 43 | Sheikh, M. M., Rezaei, M. R., & Nasseri, M. A. (2013). Heavy metals (Hg, Cr and Pb) concentrations in water and sediment of Kashaf Rood River. Toxicology *and* Environmental Health Sciences, 5(2), 65-70. | **Cr**, Hg and Pb |
| 44 | Sakan, S. M., Sakan, N. M., & Ðorđević, D. S. (2013). Trace element study in Tisa River and Danube alluvial sediment in Serbia. *International Journal of Sediment Research*, 28, 2, 234–245. | As, Cd, **Cr**, Cu, Hg, Ni, Pb, Zn, and V |
| 45 | Yu, Y., Song, J., Li, X., Yunan, H., & Li, N. (2013). Fractionation, sources and budgets of potential harmful elements in surface sediments of the East China Sea. *Marine Pollution Bulletin*, 68, 157-167. DOI 10.1016/j.marpolbul.2012.11.03 | Cd, Co, **Cr**, Cu, Ni, Pb, and V |
| 46 | Qiao, Y., Yang, Y., Gu, J., & Zhao, J. (2013). Distribution and geochemical speciation of heavy metals in sediments from coastal area suffered rapid urbanization, a case study of Shantou Bay, China. *Marine Pollution Bulletin*, 68, 140-146. DOI 10.1016/j.marpolbul.2012.12.003 | Cd, Co, **Cr**, Cu, Mn, Ni, Pb and Zn |
| 47 | Hejabi, A. T., & Basavarajappa, H. T. (2013). Heavy metals partitioning in sediments of the Kabini River in South India. Environmental Monitoring *and* Assessment, 185, 1273-1283. DOI 10.1007/s10661-012-2631-z | **Cr**, Cu, Fe, Mn, Ni, Pb and Zn |
| 48 | Massolo, S., Bignasca, A., Sarkar, S. K., Chatterjee, M., Bhattacharaya, B. D., & Alam, A. (2012). Geochemical fractionation of trace elements in sediments of Hugli River (Ganges) and Sundarban wetland (West Bengal, India). Environmental Monitoring *and* Assessment*,* 184, 7561-7577. DOI 10.1007/s10661-012-2519-y | Al, As, Cd, Co, **Cr**, Cu, Fe, Mn, Ni, Pb and Zn |
| 49 | Yang, Y., Chen, F., Zhang, L., Liu, J., Wu, S., & Kang, M. (2012). Comprehensive assessment of heavy metal contamination in sediment of the Pearl River Estuary and adjacent shelf. *Marine Pollution Bulletin*, 64, 1947-1955. DOI 10.1016/j.marpolbul.2012.04.024 | **Cr**, Cu, Ni, Pb and Zn |
| 50 | Ferraro, S. A., Curutchet, G., & Tasat, D. R. (2012). Bioaccessible heavy metals-sediment particles from Reconquista River induce lung inflammation in mice. *Environmental Toxicology and Chemistry*, 31(9), 2059-2068. DOI: 10.1002/etc.1911 | Cd, Co, **Cr**, Ni, Pb and Zn |
| 51 | Malaj, E., Rousseau, D. P. L., Laing, G. D., & Lens P. N. L. (2012). Near-shore distribution of heavy metals in the Albanian part of Lake Ohrid. Environmental Monitoring *and* Assessment, 184, 1823–1839. DOI 10.1007/s10661-011-2081-z | Co, **Cr** and Ni |
| 52 | Dundar, M. S., Altundag, H., Eyupoglu, V., Keskin, S. C., & Tutunoglu, C. (2012). Determination of heavy metals in lower Sakarya River sediments using a BCR-sequential extraction procedure. Environmental Monitoring *and* Assessment, 184, 33-41. DOI 10.1007/s10661-011-1944-7 | Cd, **Cr**, Cu, Fe, Mn, Ni, Pb, and Zn |
| 53 | Mohiuddin, K. M., Otomo, K., Ogawa, Y., Shikazono, N. (2012). Seasonal and spatial distribution of trace elements in the water and sediments of the Tsurumi River in Japan. Environmental Monitoring *and* Assessment, 184, 265–279. DOI 10.1007/s10661-011-1966-1 | Cd, Co, **Cr**, Cu, Mo, Ni, Pb and Zn |
| 54 | Shikazono, N., Tatewaki, K., Mohiuddin, K. M., Nakano, T., & Zakir H. M. (2012). Sources, spatial variation, and speciation of heavy metals in sediments of the Tamagawa River in Central Japan. *Environmental Geochemistry and Health,* 34, 13–26. DOI 10.1007/s10653-011-9409-z | **Cr**, Cu, Ni, Pb and Zn |
| 55 | Wang, S., Lin, Ch., & Cao, X. (2011). Heavy metals content and distribution in the surface sediments of the Guangzhou section of the Pearl River, Southern China. *Environmental Earth Sciences,* 64, 1593-1605. DOI 10.1007/s12665-011-1172-z | Cd, **Cr**, Cu, Pb and Zn |
| 56 | Mohiuddin, K. M., Ogawa, Y., Zakir, H. M., Otomo, K., & Shikazono, N. (2011). Heavy metals contamination in water and sediments of an urban river in a developing country. International Journal of Environmental Science *and* Technology*,* 8(4), 723-736. | As, Cd, Co, **Cr**, Cu, Ni, Pb and Zn |
| 57 | Passos, E. A., Alves, J. P. H., Garcia, C. A. B., & Costa, A. C. S. (2011). Metal fractionation in sediments of the Sergipe River, Northeast, Brazil. *Journal of the Brazilian Chemical Society,* 22(5), 828-835. | **Cr**, Cd, Cu, Ni, Pb and Zn |
| 58 | Cai, J., Cao, Y., Tan, H., Wang, Y., & Luo, J. (2011). Fractionation and ecological risk of metals in urban river sediments in Zhongshan City, Pearl River Delta. *Journal of Environmental Monitoring*, 13, 2450-2456. | Cd, **Cr**, Cu, Ni, Pb and Zn |
| 59 | Davutluoglu, O. I., Seckin, G., Ersu, C. B., Yilmaz, T., & Sari B. (2011). Heavy metal content and distribution in surface sediments of the Seyhan River, Turkey. *Journal of Environmental Management*, 92, 2250-2259. DOI 10.1016/j.jenvman.2011.04.013 | Cd, **Cr**, Cu, Mn, Ni, Pb and Zn |
| 60 | Medici, L., Bellanova, J., Belviso, C., Cavalcante, F., & Lettino, A. (2011). Trace metals speciation in sediments of the Basento River (Italy). *Applied Clay Science*, 53, 414-442. DOI 10.1016/j.clay.2010.10.029 | Cd, Co, **Cr**, Cu, Ni, Pb, Tl, V, and Zn |
| 61 | Nemati, K., Bakar, N. K. A., Abas, M.,R., & Sobhanzadeh, E. (2011). Speciation of heavy metals by modified BCR sequential extraction procedure in different depths of sediments from Sungai Buloh, Selangor, Malaysia. *Journal of Hazardous Materials*, 192, 401-410. DOI 10.1016/j.jhazmat.2011.05.039 | Cd, Co, **Cr**, Cu, Ni, Pb, V and Zn |
| 62 | Iwegbue, Ch. M. A. (2011). Chemical fractionation of metals in core sediments of Orogodo River, southern Nigeria. *Toxicological and Environmental Chemistry*, 93(7), 1341-1358. | **Cr**, Cu, Fe, Mn, Ni, Pb and Zn |
| 63 | Mayes, W. M., Jarvis, A. P., Burke, I. T., Walton, M., Feigl, V., Klebercz, O., & Gruiz, K. (2011). Dispersal and Attenuation of Trace Contaminants Downstream of the Ajka Bauxite Residue (Red Mud) Depository Failure, Hungary. Environmental Science *and* Technology, 45, 5147–5155. DOI 10.1021/es200850y | Al, As, **Cr**, Mo, Fe, Co, Ni and V |
| 64 | Wu, Y., He, M., Lin, Ch., & Fan, Z. (2011). Distribution and speciation of four heavy metals (Cd, Cr, Mn and Ni) in the surficial sediments from estuary in Daliao River and Yingkou Bay. *Environmental Earth Sciences,* 63, 163-175. DOI 10.1007/s12665-010-0680-6 | Cd, **Cr**, Mn and Ni |
| 65 | Botsou, F., Karageorgis, A. P., Dessenakis, E., & Scoullus, M. (2011). Assessment of heavy metal contamination and mineral magnetic characterization of the Asopos River sediments (Central Greece). *Marine Pollution Bulletin*, 62, 547-563. DOI 10.1016/j.marpolbul.2010.11.029 | Cd, **Cr**, Cu, Fe, Mn, Ni, Pb and Zn |
| 66 | Sundaray, S. K., Nayak, B. B., Lin, S., & Bhatta, D. (2011). Geochemical speciation and risk assessment of heavy metals in the river estuarine sediments - A case study: Mahanadi basin, India. *Journal of Hazardous Materials*, 186, 1837–1846. DOI 10.1016/j.hazmat.2010.12.081 | Co, **Cr**, Cu, Fe, Mn, Ni, Pb and Zn |
| 67 | Wu, G. H., Cao, S. S., Chen, S. R., & Cao, F. T. (2011). Accumulation and remobilization of metals in superficial sediments in Tianjin, China. Environmental Monitoring *and* Assessment, 173, 917–928. DOI 10.1007/s10661-010-1434-3 | Cd, **Cr**, Cu, Ni, Pb and Zn |
| 68 | Priadi, C., Ayrault, S., Pacini, S., & Bonte, P. (2011). Urbanization impact on metals mobility in riverine suspended sediment: Role of metal oxides. *I*nternational Journal of Environmental Science *and* Technology, 8 (1), 1-18. | Cd, **Cr**, Cu, Ni, Pb and Zn |
| 69 | Varejão, E. V. V., Bellato, C. R., Fontes, M. P. F., & Mello, J. W. V. (2011). Arsenic and trace metals in river water and sediments from the southeast portion of the Iron Quadrangle, Brazil. Environmental Monitoring *and* Assessment, 172, 631–642. DOI 10.1007/s10661-010-1361-3 | As, Cd, Co, **Cr**, Cu, Ni, Pb, and Zn |
| 70 | Yu, X., Yan, Y., & Wang, W-X. (2010). The distribution and speciation of trace metals in surface sediments from the Pearl River Estuary and the Daya Bay, Southern China. *Marine Pollution Bulletin*, 60, 1364-1371. 10.1016/j.marpolbul.2010.05.012 | **Cr**, Cu, Ni, Pb, and Zn |
| 71 | Sakan, S. M., Ðorđević, D., & Manojlović, D. D. (2010). Trace elements as tracers of environmental pollution in the canal sediments (alluvial formation of the Danube River, Serbia). Environmental Monitoring *and* Assessment*,* 167, 219-233. DOI 10.1007/s10661-009-1044-0 | As, Cd, **Cr**, Cu, Fe, Hg, Mg, Mn, Ni, Pb and Zn |
| 72 | Giridharan, L., Venugopal, T., & Jayaprakash, M. (2010). Speciation technique for the risk assessment of trace metals in the bed sediments of River Cooum, South India. *Chemical Speciation and Bioavailability*, 22( 2), 71-80. DOI 095422910X12632178514657 | **Cr**, Cu, Fe, Mn, Ni, Pb, and Zn |
| 73 | Wang, L., Yu, R., Hu, G., & Tu, X. (2010). Speciation and assessment of heavy metals in surface sediments of Jinjiang River tidal reach, southeast of China. Environmental Monitoring *and* Assessment, 165, 491-499. DOI 10.1007/s10661-009-0961-2 | Cu, Zn, Pb, **Cr**, Ni, and Cd |
| 74 | Sakan, S. M., & Ðorđević, D. (2010). Evaluation of heavy metal contamination in sediments using the method of total digestion and determination of the binding forms -Tisa River Basin, Serbia. *Journal of Environmental Science and Health Part A*, 45, 783-794. DOI: 10.1080/10934521003708893 | Cd, **Cr**, Cu, Mg, Mn, Ni, Pb and Zn |
| 75 | Kim, Y., Kim, B-K., & Kim, K. (2010). Distribution and speciation of heavy metals and their sources in Kumho River sediment, Korea. *Environmental Earth Sciences,* 60, 943-952. DOI 10.1007/s12665-009-0230-2 | Cd, Co, **Cr**, Cu, Ni, Pb and Zn |
| 76 | Beltrán, L., de la Rosa, J. D., & Santos, J. C., Beltrán M., & Gómez-Ariza J. L. (2010). Heavy metal mobility assessment in sediments from the Odiel River (Iberian Pyritic Belt) using sequential extraction. *Environmental Earth Sciences,* 61, 1493-1503. DOI 10.1007/s12665-010-0465-y | Cd, Cu, **Cr**, Fe, Hg, Mn, Ni, Pb and Zn |
| 77 | Sharmin, S., Zakir, H. M., & Shikazono, N. (2010). Fractionation profile and mobility pattern of trace metals in sediments of Nomi River, Tokyo, Japan. *Journal of Soil Science and Environmental Management*, 1(1), 001-014. | Cd, **Cr**, Cu, Fe, Mn and Ni |
| 78 | Yan, Ch., Li, Q., Zhang, X., & Li, G. (2010). Mobility and ecological risk assessment of heavy metals in surface sediments of Xiamen Bay and its adjacent areas, China. *Environmental Earth Sciences,* 60, 1469-1479. DOI 10.1007/s12665-009-0282-3 | As, Cd, Cu, **Cr**, Fe, Pb, Hg and Zn |
| 79 | Ding, H., & Ji, H. (2010). Application of chemometric methods to analyze the distribution and chemical fraction patterns of metals in sediment from a metropolitan river. *Environmental Earth Sciences,* 61, 641-657. DOI 10.1007/s12665-009-0379-8 | As, Cd, Co, **Cr**, Cu, Hg, Mn, Ni, Pb, Zn and Y |
| 80 | Alves, C. M., Boaventura, R. R. A. R., & Soares, H. M. V. M. (2009). Evaluation of Heavy Metals Pollution Loadings in the Sediments of the Ave River Basin (Portugal). *Soil and Sediment Contamination*, 18, 603-618. DOI: 10.1080/15320380903113568 | **Cr** |
| 81 | Sakan, S. M., Ðorđević, D., Manojlović, D. D., & Predrag, P. S. (2009). Assessment of heavy metal pollutants accumulation in the Tisza river sediments. *Journal of Environmental Management*, 90, 3382-3390. DOI 10.1016/j.jenvman.2009.05.013 | **Cr**, Cu, Fe, Mn, Ni, Pb and Zn |
| 82 | Liu, J., Li, Y., Zhang, B., Cao, J., Cao, Z., & Domagalski, J. (2009). Ecological risk of heavy metals in sediments of the Luan River source water. *Ecotoxicology,* 18, 748-758. DOI 10.1007/s10646-009-0345-y | As, Cd, **Cr**, Cu, Hg, Pb and Zn |
| 83 | Liu, Ch., Xu, J., Liu, Ch., Zhang, P., & Dai, M. (2009). Heavy Metals in the Surface Sediments in Lanzhou Reach of Yellow River, China. *Bulletin of* Environmental *Contamination and* Toxicology, 82, 26-30. DOI 10.1007/s00128-008-9563-x | As, **Cr**, Cu, Mn, Pb and Zn |
| 84 | Rath, P., Panda, U. C., Bhatta, D., & Sahu, K. C. (2009). Use of sequential leaching, mineralogy, morphology and multivariate statistical technique for quantifying metal pollution in highly polluted aquatic sediments - A case study: Brahmani and Nandira Rivers, India. *Journal of Hazardous Materials*, 163, 632-644. DOI 10.1016/j.hazmat.2008.07.048 | Cd, Co, **Cr**, Cr, Fe, Mn, Ni, Pb and Zn |
| 85 | Lesven, L., Lourino-Cabana, B., Billon, G., Proix, N., Recourt, P., Ouddane, B., Fischer, J. C., Boughriet, A. (2009). Water-Quality Diagnosis and Metal Distribution in a Strongly Polluted Zone of Deûle River (Northern France). Water, Air, & Soil Pollution, 198, 31-44. DOI 10.1007/s11270-008-9823-8 | **Cr**, Cd, Cu, Ni, Pb and Zn |
| 86 | Malferrari, D., Brigatti, M. F., Laurora, A., & Pini, S. (2009). Heavy metals in sediments from canals for water supplying and drainage: Mobilization and control strategies. *Journal of Hazardous Materials*, 161, 723-729. DOI 10.1016/j.hazmat.2008.04.014 | As, Cd, **Cr**, Hg, Ni, Pb and Zn |
| 87 | Vieira, J. S., Botelho, C. M. S., & Boaventura, R. A. R. (2009). Trace Metal Fractionation by the Sequential Extraction Method in Sediments from the Lis River (Portugal). *Soil and Sediment Contamination*, 18, 102-119. DOI: 10.1080/15320380802304359 | Al, **Cr**, Cu, Fe, Mn, Ni, Pb and Zn |
| 88 | Zakir, H. M., Shikazono, N., & Otomo, K. (2008). Geochemical Distribution of Trace Metals and Assessment of Anthropogenic Pollution in Sediments of Old Nakagawa River, Tokyo, Japan. *American Journal of Environmental Science*, 4 (6), 654-665. | Cd, **Cr**, Cu, Pb and Zn |
| 89 | Lasheen, M. R.,& Ammar, N. S. (2009). Speciation of some heavy metals in River Nile sediments, Cairo, Egypt. *Environmentalist*, 29, 8-16. DOI 10.1007/s10669-008-9175-3 | Cd, **Cr**, Cu, Fe, Mn, Ni, Pb and Zn |
| 90 | Jain, C. K., Gupta, H., & Chakrapani, G. J. (2008). Enrichment and fractionation of heavy metals in bed sediments of River Narmada, India. Environmental Monitoring *and* Assessment, 141, 35-47. DOI 10.1007/s10661-007-9876-y | Cd, **Cr**, Cu, Fe, Mn, Ni, Pb and Zn |
| 91 | Morillo, J., Usero, J., & Rojas, R. (2008). Fractionation of metals and As in sediments from a biosphere reserve (Odiel salt marshes) affected by acidic mine drainage. Environmental Monitoring *and* Assessment, 139, 329-337. DOI 10.1007/s10661-007-9839-3 | As, Cd, **Cr**, Cu, Fe, Mn, Ni, Pb and Zn |
| 92 | Arias, R., Barona, A., Ibarra-Berastegi, B., Aranguiz, I., & Elías, A. (2008). Assessment of metal contamination in dregded sediments using fractionation and Self-Organizing Maps. *Journal of Hazardous Materials*, 151, 78-85. DOI 10.1016/j.hazmat.2007.05.048 | Ca, Co, **Cr**, Cu, Fe, K, Mg, Mn, Na, Ni, Pb and Zn |
| 93 | Álvarez, M. B., Garrido, M., Lista, A. G., & Fernández Band, B. S. (2008). Three-way multivariate analysis of metal fractionation results from sediment samples obtained by different sequential extraction procedures and ICP-OES. *Analytica Chimica Acta*, 620, 34-43. DOI 10.1016/j.aca.2008.05.035 | Cd, **Cr,** Cu, Pb, and Zn |
| 94 | Iwegbue, Ch. M. A., Eghwrudje, M. O., Nwajei G. E., & Egboh S. H. O. (2007). Chemical speciation of heavy metals in the Ase River sediment, Niger Delta, Nigeria. *Chemical Speciation and Bioavailability*, 19(3), 117-127. DOI 10.3184/095422901782775453A | Cd, **Cr**, Cu, Fe, Mn, Ni, Pb and Zn |
| 95 | Li, Q., Wu, Z., Chu, B., Zhang, N., Cai, S., & Fang, J. (2007). Heavy metals in coastal wetland sediments of the Pearl River Estuary, China. *Environmental Pollution*, 149, 158-164. DOI 10.1016/j.envpol.2007.01.006 | Cd, **Cr,** Cu, Ni, Pb and Zn |
| 96 | Talebi, S. M., & Semnani, A. (2007). Four-Stage Sequential Extraction of Heavy Metals from Zayandeh-Rud River Sediments. *Asian Journal of Chemistry,* 19(2), 1553-1558. | **Cr**, Cu, Fe, Mn, Ni, Pb and Zn |
| 97 | Kolowski Rodrigues, M. L., & Formoso, M. L. L. (2006). Geochemical distribution of selected heavy metals in stream sediments affected by tannery activities. Water, Air, & Soil Pollution, 169, 167-184. | Al, Cd, **Cr**, Cu, Fe, Mn, Ni, Ti and Zn |
| 98 | Olivares-Rieumont, S., de la Rosa, D., Lima, L., Graham, D. W., D’ Alessandro, K., Borroto, J., Martínez, F., & Sánchez, J. (2005). Assessment of heavy metal levels in Almendares River sediments - Havana City, Cuba. *Water Research*, 39, 3945-3953. DOI 10.1016/j.watres.2005.07.011 | Cd, Co, **Cr**, Cu, Pb and Zn |
| 99 | Reis, A. R., Parker, A., Carter, J., & Portugal, M. (2005). Distribution of selected heavy metals in sediments of the Águeda River (Central Portugal). *Journal of Environmental Science and Health, Part A*, A40, 305-316. DOI: 10.1081/ESE-200045535 | Cd, Co, **Cr**, Cu, Fe, Mn, Ni, Pb and Zn |
| 100 | Głosińska, G., Sobczyński, T., Boszke, L., Bierła, K., & Siepak, J. (2005). Fractionation of some heavy metals in bottom sediments from the middle Odra River (Germany/Poland). *Polish Journal of Environmental Studies*, 14(3), 305-317. | Cd, **Cr**, Cu, Fe, Mn, Ni, Pb and Zn |
| 101 | Singh, K. P., Mohan, D., Singh V. K., & Malik, A. (2005). Studies on distribution and fractionation of heavy metals in Gomti river sediments - a tributary of the Ganges, India. *Journal of Hydrology*, 312, 14-27. DOI 10.1016/j.jhydrol.2005.01.021 | Cd, **Cr**, Cu, Fe, Mn, Ni and Pb |
| 102 | Filgueiras, A.V., Lavilla, I., & Bendicho, C. (2004). Evaluation of distribution, mobility and binding behaviour of heavy metals in surficial sediments of Louro River (Galicia, Spain) using chemometric analysis: a case study. *Science of the total Environment*, 330, 115-129. DOI 10.1016/j.scitotenv.2004.03.038 | Pb, Cd, **Cr**, Cu and Ni |
| 103 | Almeida, C.M.R., Mucha A.P., Vasconcelos M.T.S.D. (2004). Influence of the Sea Rush Juncus maritimuson Metal Concentration and Speciation in Estuarine Sediment Colonized by the Plant. *Environmental Science & Technology*, 38, 3112-3118. | Cd, **Cr**, Cu, Fe, Mn, Ni, Pb and Zn |
| 104 | Morillo, J., Usero, J., & Gracia, I. (2004). Heavy metal distribution in marine sediments from the southwest coast of Spain. *Chemosphere*, 55, 431-442. DOI 10.1016/j.chemosphere.2003.10.047 | Cd, **Cr**, Cu, Fe, Mn, Ni and Zn |
| 105 | Sáenz, W., Blasco, J., & Gómez-Parra, A. (2003). Speciation of heavy metals in recent sediments of three coastal ecosystems in the Gulf of Cádiz, southwest Iberian Peninsula. *Environmental Toxicology and Chemistry*, 22(12), 2833-2839. | Cd, **Cr**, Cu, Fe, Mn, Pb and Zn |
| 106 | Tsai, L-J., Yu, K-Ch., Chen, S-F., & Kung, P-Y. (2003). Effect of temperature on removal of heavy metals from contaminated river sediments via bioleaching. *Water Research*, 37, 2449-2457. | Co, **Cr**, Cu, Ni, Pb and Zn |
| 107 | Peng, S-H., Wang, W-X., & Chen, J. (2003). Partitioning of trace metals in suspended sediments from Huanghe and Changjiang rivers in Eastern China. Water, Air, & Soil Pollution*,* 148, 243-258. | Cd, **Cr**, and Zn |
| 108 | Tsai, L. J., Yu, K-Ch., Chen, S-F., Kung, P-Y., Chang, Ch-Y., & Lin, Ch-H. (2003). Partitioning variation of heavy metals in contaminated river sediment via bioleaching: effect of sulfur added to total solids ratio. *Water Research*, 37, 4623-4630. | **Cr**, Cu, Ni, Pb and Zn |
| 109 | Galán, E., Gómez-Ariza, J. L., González, I., Fernández-Celiani, J. C., Morales, E., & Giráldez, I. (2003). Heavy metal partitioning in river sediments severely polluted by acid mine drainage in the Iberian Pyrite Belt. *Applied Geochemistry*, 18, 409-421. | As, Cd, **Cr**, Cu, Fe, Mn, Ni, Pb and Zn |
| 110 | Akcay, H., Oguz, A., & Karapire, C. (2003). Study of heavy metal pollution and speciation in Buyak Menderes and Gediz river sediments. *Water Research*, 37, 813-822. | Co, Cr, Cu, Fe, Mn, Ni, Pb and Zn |
| 111 | Morillo, J., Usero, J., & Gracia, I. (2002). Partitioning of metals in sediments from the Odiel River (Spain). *Environment International,* 28, 263-271. | Cd, Co, **Cr**, Cu, Fe, Ni, Pb and Zn |
| 112 | Balkis, N., & Çaĝatay, M. N. (2001). Factors controlling metal distribution in the surface sediments of the Erdek Bay, Sea of Marmara, Turkey. *Environment International*, 27, 1-13. | **Cr**, Cu, Fe, Hg, Mn, Pb and Zn |
| 113 | Birch, G., Siaka, M., & Owens, Ch. (2001). The source of anthropogenic heavy metals in fluvial sediments of a rural catchment: Coxs River, Australia. Water, Air, & Soil Pollution, 126, 13-35. | Cd, Co, **Cr**, Cu, Fe, Mn, Ni, Pb and Zn |
| 114 | Ho, T. L. T., & Egashira, K. (2000). Heavy metal characterization of river sediment in Hanoi, Vietnam. *Communications in Soil Science and Plant Analysis,* 31, 17-18, 2901-2916. | Cd, **Cr**, Cu, Ni, Pb and Zn |
| 115 | Helios-Rybicka, E., & Wilson, M. J. (2000). Direct and Indirect Methods for Chromium Identification in Industrial Wastes. *Environmental Science and Pollution Research*, 7(1), 7-13. | **Cr**, Fe and Zn |
| 116 | González, A. E., Rodrioguez, M. T., Sánchez, J. C., Espinosa, J. F., & De La Rosa, F. J. B. (2000). Assessment of metals in sediments in a tributary of Guadalquivir River (Spain). Heavy metal partitioning and relation between the water and sediment system. Water, Air, & Soil Pollution, 121, 11-29. | Cd, **Cr**, Cu, Pb and Zn |
| 117 | Rate, A. W., Robertson, A. E., & Borg, A. T. (2000). Distribution of heavy metals in near-shore sediments of the Swan River Estuary, Western Australia. Water, Air, & Soil Pollution, 124, 155-168. | **Cr**, Cu, Pb and Zn |
| 118 | Gómez-Ariza, J. L., Giraldez, I., Sánchez-Rodas, D., & Morales, E. (2000). Comparison of the feasibility of three extraction procedures for trace metal partitioning in sediments from south-west Spain. *The Science of the Total Environment*, 246, 271-283. | As, Cd, **Cr**, Cu, Fe, Hg, Mn, Ni, Pb and Zn |

**Table 2S** The results of determined and certified values for certified reference material (BCR 701), mean (mg/kg dry weight ± SD), n = 6

| Step | Chromium fraction | Certified value (mg/kg) | Determined value (mg/kg) | % Recovery |
| --- | --- | --- | --- | --- |
| 1 | F(1) - weak acid soluble fraction | 2.26 ± 0.16 | 2.19 ± 0.25 | 96.9 |
| 2 | F(2) - reducible fraction | 45.7 ± 2.0 | 43.5 ± 1.7 | 95.2 |
| 3 | F(3) - oxidizable fraction | 143 ± 7 | 149.0 ± 4.0 | 104 |
| 4 | F(4) - residual fraction | 62.5 ± 7.4 ^a^ | 64.3 ^b^ | 103 |
| 5 | Cr-tot. | 272 ± 20 ^a^ | 259 ± 18 | 95.2 |
| ^a^ Value given by producer as uncertified  ^b^ Fraction F(4)-Cr was calculated as the difference between the total content of chromium and the sum of the fractions extracted during the previous steps: Cr-tot. – [F(1)-Cr + F(2)-Cr + F(3)-Cr] | | | | |

**Table 3S** Effect of sediment sample preparation on the determination results of chemical fractions of chromium (A – frozen raw sample; B - air dried raw sample; C – air dried ground sample; D - oven dried ground sample). Critical value of Student's *t* distribution with *4* degrees of freedom *t* = 2.776, (two-tailed test, P = 95*%*)

| Sample | Cr-pseudototal | | F(1)-Cr | F(2)-Cr | | F(3)-Cr | | F(4)-Cr | |
| --- | --- | --- | --- | --- | --- | --- | --- | --- | --- |
|  | (mg/kg) ± SD | *t_c_* | (mg/kg) | (mg/kg) ± SD | *t_c_* | (mg/kg) ± SD | *t_c_* | (mg/kg) ± SD | *t_c_* |
| P-1A | 2.48 ± 0.40 |  | < LOD | 0.17 ± 0.03 |  | 0.81 ± 0.03 |  | 0.16 ± 0.01 |  |
|  |  | 0.079 |  |  | 0.4805 |  | 0.420 |  | 1.225 |
| P-1B | 2.46 ± 0.28 |  | < LOD | 0.18 ± 0.02 |  | 0.78 ± 0.12 |  | 0.15 ± 0.01 |  |
|  |  | 0.292 |  |  | **8.913** |  | 0.750 |  | 1.640 |
| P-1C | 2.40 ± 0.22 |  | < LOD | 0.41 ± 0.04 |  | 0.70 ± 0.14 |  | 0.18 ± 0.03 |  |
|  |  | 0.989 |  |  | 0.775 |  | 0.141 |  | 0.480 |
| P-1D | 2.64 ± 0.20 |  | < LOD | 0.43 ± 0.02 |  | 0.68 ± 0.10 |  | 0.19 ± 0.02 |  |
|  |  |  |  |  |  |  |  |  |  |
|  |  |  |  |  |  |  |  |  |  |
| P-2A | 29.2 ± 3.6 |  | < LOD | 2.48 ± 0.27 |  | 21.3 ± 2.8 |  | 2.50 ± 0.27 |  |
|  |  | 0.063 |  |  | 0.235 |  | 0.05 |  | 0.439 |
| P-2B | 29.0 ± 4.2 |  | < LOD | 2.43 ± 0.13 |  | 21.4 ± 2.6 |  | 2.39 ± 0.34 |  |
|  |  | 0.251 |  |  | **29.12** |  | **4.00** |  | 0.623 |
| P-2C | 28.3 ± 2.4 |  | < LOD | 9.34 ± 0.39 |  | 14.9 ± 1.1 |  | 2.53 ± 0.19 |  |
|  |  | 0.147 |  |  | 0.797 |  | 0.92 |  | 0.451 |
| P-2D | 28.6 ± 2.6 |  | < LOD | 9.02 ± 0.30 |  | 15.8 ± 1.3 |  | 2.46 ± 0.19 |  |
|  |  |  |  |  |  |  |  |  |  |
|  |  |  |  |  |  |  |  |  |  |
| P-3A | 121 ± 9 |  | < LOD | 2.21 ± 0.30 |  | 106 ± 7 |  | 8.58 ± 0.43 |  |
|  |  | 0.144 |  |  | 2.980 |  | 0.19 |  | 0.962 |
| P-3B | 120 ± 8 |  | < LOD | 2.94 ± 0.30 |  | 105 ± 6 |  | 8.17 ± 0.60 |  |
|  |  | 0.367 |  |  | **13.36** |  | **4.04** |  | **5.727** |
| P-3C | 122 ± 5 |  | < LOD | 22.4 ± 2.60 |  | 90.4 ± 1.8 |  | 4.81 ± 0.82 |  |
|  |  | 0.271 |  |  | 1.362 |  | 0.62 |  | 0.276 |
| P-3D | 123 ± 4 |  | < LOD | 24.8 ± 1.60 |  | 89.2 ± 2.8 |  | 4.98 ± 0.68 |  |
|  |  |  |  |  |  |  |  |  |  |
|  |  |  |  |  |  |  |  |  |  |
| P-4A | 204 ± 23 |  | < LOD | 15.8 ± 2.1 |  | 163 ± 16 |  | 11.1 ± 0.4 |  |
|  |  | 0.102 |  |  | 0.479 |  | 0.26 |  | 2.402 |
| P-4B | 206 ± 25 |  | < LOD | 14.6 ± 3.8 |  | 160 ± 12 |  | 12.1 ± 0.6 |  |
|  |  | 0.057 |  |  | **11.42** |  | **4.27** |  | **8.637** |
| P-4C | 207 ± 17 |  | < LOD | 60.9 ± 5.9 |  | 123 ± 9 |  | 8.93 ± 0.21 |  |
|  |  | 0.321 |  |  | 1.365 |  | 1.15 |  | 2.267 |
| P-4D | 202 ± 21 |  | < LOD | 55.1 ± 44 |  | 131 ± 8 |  | 8.22 ± 0.50 |  |
|  |  |  |  |  |  |  |  |  |  |
|  |  |  |  |  |  |  |  |  |  |
| P-5A | 83.8 ± 8.2 |  | < LOD | 5.87 ± 0.55 |  | 68.1 ± 4.6 |  | 5.01 ± 0.32 |  |
|  |  | 0.229 |  |  | 1.763 |  | 0.75 |  | 1.109 |
| P-5B | 82.4 ± 6.7 |  | < LOD | 5.12 ± 0.49 |  | 65.3 ± 4.5 |  | 5.39 ± 0.50 |  |
|  |  | 0.464 |  |  | **19.46** |  | **4.17** |  | **4.057** |
| P-5C | 84.9 ± 6.5 |  | < LOD | 25.0 ± 1.7 |  | 52.4 ± 2.9 |  | 4.07 ± 0.26 |  |
|  |  | 0.096 |  |  | 0.872 |  | 0.83 |  | 1.587 |
| P-5D | 85.3 ± 3.2 |  | < LOD | 23.6 ± 2.2 |  | 54.3 ± 2.7 |  | 3.72 ± 0.28 |  |
|  |  |  |  |  |  |  |  |  |  |
|  |  |  |  |  |  |  |  |  |  |
| P-6A | 233 ± 15 |  | < LOD | 17.3 ± 2.3 |  | 171 ± 12 |  | 15.3 ± 1.3 |  |
|  |  | 0.517 |  |  | 0.925 |  | 0.24 |  | 1.457 |
| P-6B | 226 ± 18 |  | < LOD | 15.6 ± 2.2 |  | 169 ± 8 |  | 17.1 ± 1.7 |  |
|  |  | 0.673 |  |  | **17.61** |  | **3.12** |  | **8.625** |
| P-6C | 234 ± 10 |  | < LOD | 48.7 ± 2.4 |  | 152 ± 5 |  | 8.38 ± 0.42 |  |
|  |  | 0.503 |  |  | 0.935 |  | 0.44 |  | 2.391 |
| P-6D | 239 ± 14 |  | < LOD | 47.2 ± 1.4 |  | 154 ± 6 |  | 9.23 ± 0.45 |  |
| SD standard deviation, LOD limit of detection  The values of *t_c_* > 2.776 marked in bold | | | | | | | | | |

**Table 4S** Effect of sediment sample preparation on the determination results of carbonate and oxide fractions of Ca, Fe and Mn (A – frozen raw sample; B - air dried raw sample; C – air dried ground sample; D - oven dried ground sample). Critical value of Student's *t* distribution with *4* degrees of freedom *t* = 2.776 (two-tailed test, P = 95%)

| Sample | | F(2)-Ca | | F(3)-Ca | | F(2)-Fe | | F(3)-Fe | | F(2)-Mn | | F(3)-Mn | |  |
| --- | --- | --- | --- | --- | --- | --- | --- | --- | --- | --- | --- | --- | --- | --- |
|  |  | (mg/kg) | *t_c_* | (mg/kg) | *t_c_* | (mg/kg) | *t_c_* | (mg/kg) | *t_c_* | (mg/kg**)** | *t_c_* | **(**mg/kg) | *t_c_* |  |
| P-1A | 146 ± 17 |  | 62.6 ± 9.1 |  | 71.7 ± 5.9 |  | 1030 ± 72 |  | 14.1 ± 0.7 |  | 17.3 ± 2.1 |  |  |  |
|  |  | 0.543 |  | 0.231 |  | **4.251** |  | 0.380 |  | **2.838** |  | **3.160** |  |  |
| P-1B | 154 ± 19 |  | 61.1± 6.6 |  | 56.4 ± 2.0 |  | 1049 ± 48 |  | 12.1 ± 1.0 |  | 21.4 ± 0.8 |  |  |  |
|  |  | 0.462 |  | **3.502** |  | **40.14** |  | 1.408 |  | **14.73** |  | **12.79** |  |  |
| P-1C | 148 ± 12 |  | 75.3 ± 2.4 |  | 430 ± 15 |  | 1120 ± 73 |  | 28.1 ± 1.6 |  | 12.5 ± 0.9 |  |  |  |
|  |  | 0.333 |  | 1.048 |  | 0.213 |  | 0.856 |  | 0.166 |  | 0.540 |  |  |
| P-1D | 145 ± 10 |  | 79.1 ± 5.8 |  | 433 ± 19 |  | 1070 ± 70 |  | 27.8 ± 2.7 |  | 11.9 ± 1.7 |  |  |  |
|  |  |  |  |  |  |  |  |  |  |  |  |  |  |  |
|  |  |  |  |  |  |  |  |  |  |  |  |  |  |  |
| P-2A | 501 ± 40 |  | 133 ± 15 |  | 171 ± 12 |  | 1560 ± 190 |  | 41.5 ± 2.5 |  | 65.9 ± 5.4 |  |  |  |
|  |  | 0.551 |  | 0.148 |  | **3.251** |  | 0.220 |  | **3.467** |  | **3.001** |  |  |
| P-2B | 486 ± 25 |  | 131 ± 18 |  | 144 ± 8 |  | 1590 ± 140 |  | 34.7 ± 2.3 |  | 79.5 ± 5.7 |  |  |  |
|  |  | 1.835 |  | 0.082 |  | **32.25** |  | 2.043 |  | **30.38** |  | **15.68** |  |  |
| P-2C | 525 ± 27 |  | 132 ± 11 |  | 716 ± 29 |  | 1380 ± 110 |  | 101 ± 3 |  | 23.8 ± 2.3 |  |  |  |
|  |  | 0.842 |  | 0.186 |  | 0.442 |  | 0.573 |  | 0.672 |  | 0.667 |  |  |
| P-2D | 493 ± 60 |  | 130 ± 15 |  | 708 ± 12 |  | 1340 ± 50 |  | 99.6 ± 2.0 |  | 22.6 ± 2.1 |  |  |  |
|  |  |  |  |  |  |  |  |  |  |  |  |  |  |  |
|  |  |  |  |  |  |  |  |  |  |  |  |  |  |  |
| P-3A | 1510 ± 110 |  | 233 ± 23 |  | 238 ± 16 |  | 1250 ± 70 |  | 14.9 ± 1.4 |  | 5.51 ± 0.15 |  |  |  |
|  |  | 0.228 |  | 0.137 |  | **4.660** |  | 0.890 |  | **3.480** |  | **16.15** |  |  |
| P-3B | 1490 ± 105 |  | 229 ± 45 |  | 184 ± 12 |  | 1290 ± 34 |  | 11.3 ± 1.1 |  | 8.45 ± 0.21 |  |  |  |
|  |  | 1.541 |  | 1.353 |  | **33.27** |  | **4.712** |  | **5.958** |  | **6.124** |  |  |
| P-3C | 1590 ± 40 |  | 193 ± 10 |  | 553 ± 15 |  | 1100 ± 61 |  | 16.9 ± 1.2 |  | 9.66 ± 0.27 |  |  |  |
|  |  | 0.215 |  | 0.615 |  | 0.076 |  | 1.558 |  | 0.939 |  | 0.355 |  |  |
| P-3D | 1610 ± 156 |  | 200 ± 17 |  | 554 ± 17 |  | 1040 ± 27 |  | 15.9 ± 1.4 |  | 9.78 ± 0.52 |  |  |  |
|  |  |  |  |  |  |  |  |  |  |  |  |  |  |  |
|  |  |  |  |  |  |  |  |  |  |  |  |  |  |  |
| P-4A | 2802 ± 160 |  | 157 ± 11 |  | 92.4 ± 8.9 |  | 640 ± 55 |  | 8.82 ± 0.68 |  | 7.84 ± 0.60 |  |  |  |
|  |  | 0.097 |  | 1.291 |  | **4.120** |  | 0.629 |  | **5.206** |  | **5.909** |  |  |
| P-4B | 2790 ± 143 |  | 176 ± 23 |  | 68.9 ± 4.3 |  | 667 ± 50 |  | 6.69 ± 0.20 |  | 10.3 ± 0.4 |  |  |  |
|  |  | 1.130 |  | **9.616** |  | **39.32** |  | 2.618 |  | **8.004** |  | **3.819** |  |  |
| P-4C | 2930 ± 160 |  | 46.1 ± 4.3 |  | 316 ± 10 |  | 761 ± 37 |  | 10.5 ± 0.8 |  | 8.71 ± 0.6 |  |  |  |
|  |  | 0.381 |  | **3.766** |  | 1.221 |  | 1.187 |  | 1.468 |  | 1.309 |  |  |
| P-4D | 2886 ± 120 |  | 61.8 ± 5.8 |  | 327 ± 12 |  | 730 ± 26 |  | 11.4 ± 0.7 |  | 8.12 ± 0.50 |  |  |  |
|  |  |  |  |  |  |  |  |  |  |  |  |  |  |  |
|  |  |  |  |  |  |  |  |  |  |  |  |  |  |  |
| P-5A | 530 ± 45 |  | 498 ± 42 |  | 189 ± 11 |  | 1230 ± 128 |  | 9.32 ± 0.70 |  | 14.5 ± 1.2 |  |  |  |
|  |  | 0.343 |  | 0.544 |  | **4.430** |  | 0.630 |  | **5.172** |  | 1.597 |  |  |
| P-5B | 514 ± 67 |  | 477 ± 52 |  | 151 ± 10 |  | 1290 ± 104 |  | 6.90 ± 0.41 |  | 16.2 ± 1.4 |  |  |  |
|  |  | **8.267** |  | **12.67** |  | **23.39** |  | 2.497 |  | **16.64** |  | **4.724** |  |  |
| P-5C | 894 ± 43 |  | 92.0± 8.1 |  | 342 ± 10 |  | 1080 ± 102 |  | 14.7 ± 0.7 |  | 10.8 ± 1.4 |  |  |  |
|  |  | 0.432 |  | 2.153 |  | 1.550 |  | 0.534 |  | 1.691 |  | 0.208 |  |  |
| P-5D | 880 ± 36 |  | 108 ± 10 |  | 328 ± 12 |  | 1120 ± 80 |  | 13.8 ± 0.6 |  | 10.6 ± 0.9 |  |  |  |
|  |  |  |  |  |  |  |  |  |  |  |  |  |  |  |
|  |  |  |  |  |  |  |  |  |  |  |  |  |  |  |
| P-6A | 7230 ± 200 |  | 2414 ± 186 |  | 439 ± 36 |  | 3360 ± 410 |  | 157 ± 10 |  | 72.7± 3.9 |  |  |  |
|  |  | 0.235 |  | 0.415 |  | **10.18** |  | 0.390 |  | **3.091** |  | **7.697** |  |  |
| P-6B | 7280 ± 310 |  | 2363 ± 104 |  | 207 ± 17 |  | 3550 ± 390 |  | 133 ± 9 |  | 98.5 ± 4.3 |  |  |  |
|  |  | **6.475** |  | **21.85** |  | **29.19** |  | **2.441** |  | **6.890** |  | **6.476** |  |  |
| P-6C | 8867 ± 290 |  | 902 ± 151 |  | 878 ± 36 |  | 4280 ± 340 |  | 174 ± 5 |  | 70.0 ± 4.5 |  |  |  |
|  |  | 0.125 |  | 0.168 |  | 0.130 |  | 0.102 |  | 0.184 |  | 0.560 |  |  |
| P-6D | 8890 ± 130 |  | 910 ± 165 |  | 881 ± 713 |  | 4310 ± 380 |  | 175 ± 8 |  | 72.4 ± 5.9 |  |  |  |
| The values of *t_c_* > 2.776 marked in bold | | | | | | | | | | | | | | |

1. Corresponding author. Phone: +48 48 3617517; fax: +48 48 3617598.

   E-mail address: r.swietlik@uthrad.pl (R. Świetlik). [↑](#footnote-ref-1)
